# Variations in brachial plexus and the relationship of median nerve with the axillary artery: a case report

**DOI:** 10.1186/1749-7221-2-21

**Published:** 2007-10-03

**Authors:** Suruchi Singhal, Vani Vijay Rao, Roopa Ravindranath

**Affiliations:** 1 Department of Anatomy, St John's Medical College, Bangalore – 560034, India

## Abstract

**Background:**

Brachial Plexus innervates the upper limb. As it is the point of formation of many nerves, variations are common. Knowledge of these is important to anatomists, radiologists, anesthesiologists and surgeons. The presence of anatomical variations of the peripheral nervous system is often used to explain unexpected clinical signs and symptoms.

**Case Presentation:**

On routine dissection of an embalmed 57 year old male cadaver, variations were found in the formation of divisions and cords of the Brachial Plexus of the right side. Some previously unreported findings observed were; direct branches to the muscles Pectoralis Minor and Latissimus dorsi from C6, innervation of deltoid by C6 and C7 roots and the origin of lateral pectoral nerve from the posterior division of upper trunk. The median nerve was present lateral to axillary artery. The left side brachial plexus was also inspected and found to have normal anatomy.

**Conclusion:**

The probable cause for such variations and their embryological basis is discussed in the paper. It is also concluded that although these variations may not have affected the functioning of upper limb in this individual, knowledge of such variations is essential in evaluation of unexplained sensory and motor loss after trauma and surgical interventions to the upper limb.

## Background

The brachial plexus is usually formed by the fusion of the anterior primary rami of the C5-8 and the T1 spinal nerves. It supplies the muscles of the back and the upper limb. The C5 and C6 fuse to form the upper trunk, the C7 continues as the middle trunk and the C8 and T1 join to form the lower trunk. Each trunk, soon after its formation, divides into anterior and posterior divisions. The anterior divisions of the upper and middle trunk**s **form the lateral cord, the anterior division of the lower trunk continues as the medial cord and the posterior divisions of all three form the posterior cord. The cords then give rise to various branches that form the peripheral nerves of the upper limb. The anterior divisions supply the flexor compartments of upper limb and the posterior divisions, the extensor compartments. Since the brachial plexus is a complex structure, variations in formation of roots, trunks, divisions and cords are common. The present study deals with some of the common variations and some hitherto unknown variations of the brachial plexus.

Axillary artery passes between the lateral and medial cords of the plexus. The medial root of median nerve crosses the axillary artery to unite with the lateral root to form the median nerve which is lateral and anterior to the axillary artery.

## Case presentation

The study was done in the Department of Anatomy, St. John's Medical College, Bangalore. On routine dissection of an embalmbed 57 year old male cadaver, variations in the formation of the Brachial plexus of the right side were found. The clavicle and the scalenus anterior were cut to expose the roots and trunks of the plexus. The divisions and their branches were followed to the muscle they supplied for confirmation. The left side brachial plexus was also inspected and was found to be normal.

**Figure 1:**
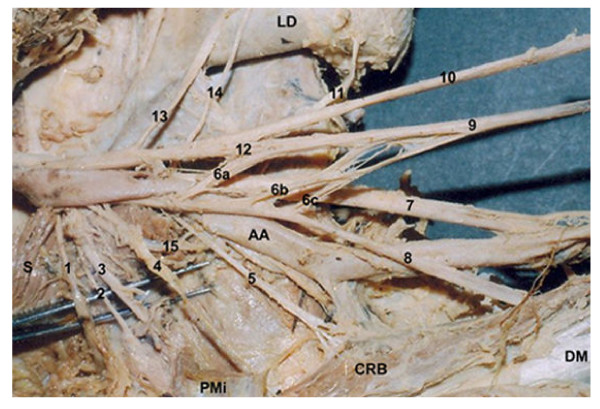
Brachial Plexus of the right side of 57 year old male cadaver (In Situ). 1. Suprascapular Nerve, 2. ? Upper Subscapular Nerve, 3. Nerve to Pectoralis Minor, 4. Nerve to Deltoid, 5. Nerve to Coracobrachialis, 6 a, b, c. Lateral Roots of the Median Nerve, 7. Ulnar Nerve, 8. Musculocutaneous Nerve, 9. Median Nerve, 10. Radial nerve, 11. Nerve to Latisimus Dorsi, 12. Medial Root of the Median nerve, 13. Long Thoracic Nerve, 14. ? Lower Subscapular Nerve (Cut), 15. Axillary Nerve, LD. Latisimus Dorsi Muscle, S. Subscapularis Muscle, CRB Coracobrachialis Muscle, DM. Deltoid Muscle, AA. Axillary Artery.

**Figure 2:**
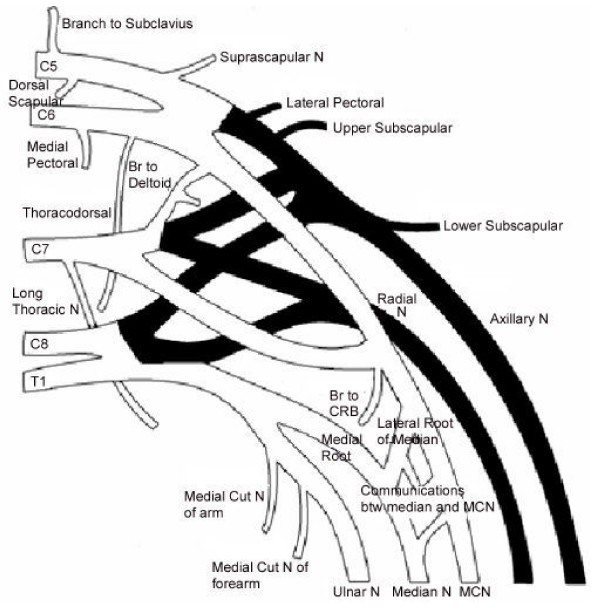
Schematic Diagram of the Brachial Plexus of the right side of 57 year old male cadaver. Shaded portions represent the posterior cord and its branches.

The brachial plexus was formed from roots C5, C6, C7, C8 and T1 (Figure 1 and 2). The upper trunk was formed by the union of C5 and C6. Before joining the C6, the C5 gave a direct branch to the Subclavius Muscle and the Dorsal scapular Nerve. Similarily the C6 gave two small direct branches to Pectoralis Minor and a large branch to the Latissimus Dorsi Muscle (Thoracodorsal Neve).

The upper trunk after its formation gave the Suprascapular nerve and then divided into an anterior division and a posterior division. The posterior division gave 2 branches to the Subscapularis, a branch to Pectoralis major and then fused with the posterior branches of middle and lower trunks. This fused portion was the posterior cord lying posterior to the axillary artery and it gave one branch to subscapularis and continued as the Axillary nerve. The anterior division of the upper trunk gave a branch that joined with the anterior division of the middle trunk to supply the deltoid muscle. It then joined the anterior division of middle trunk completely to form the lateral cord that lay lateral to the second part of axillary artery. The lateral cord gave rise to a direct branch to the coracobrachialis, the lateral root of the median nerve and thereafter continued as the musculocutaneous nerve. The musculocutaneous nerve gave two communicating branches to the median nerve and the lateral root gave a communicating branch to the first communicating branch of the median nerve.

The middle trunk gave a thin branch that fused with a branch of the anterior division of upper trunk to supply the deltoid muscle. It then gave the Long Thoracic Nerve that supplied the Serratus Anterior muscle. It then gave rise to one anterior division and two posterior divisions. The anterior division fused with the anterior division of the upper trunk. One of the posterior divisions fused with the posterior divisions of the upper trunk and the lower trunk. The second posterior division was the largest and it formed the radial nerve which was joined by the smaller posterior division of lower trunk.

The lower trunk divided into one anterior and two posterior divisions. The first fused with the posterior divisions of middle trunk and the upper trunk. The second joined the radial nerve. The anterior division, which was the medial cord, gave rise to the medial root of the median, medial cutaneous nerve of the arm, the medial cutaneous nerve of forearm and continued as the ulnar nerve. The medial cord was medial to the axillary artery (Table 1).

**Table 1:** Branches from the Brachial Plexus

Branches from Roots	Branches from Trunks	Branches from fusion of Trunks
**Direct Branches**	**Branches from the Upper trunk**	**Branches from fusion of upper and middle trunks**
Nerve to Subclavius (C5) Dorsal Scapular Nerve (C5) Nerve to Pectoralis Minor ?Medial pectoral (C6) Thoracodorsal Nerve (C6) Long Thoracic Nerve (C7) Nerve to deltoid muscle (C7)	Suprascapular nerve (C5, C6) Branches to subscapularis ?Upper Subscapular (C5, C6) Branch to Pectoralis Major ?Lateral pectoral (C5, C6) Nerve to deltoid muscle (C6)	Branch to Deltoid Muscle (C5,C6, C7) Nerve to Coracobrachialis (C5,C6, C7) Lateral root of the Median Nerve (C5,C6, C7) Musculocutaneous nerve (C5,C6, C7)
	**Branches from the Middle Trunk**	**Branches from fusion of middle and lower trunks**
	Branch to deltoid (C7) Radial Nerve (C7)	Radial nerve (C7,C8, T1)
	**Branches from the Lower Trunk**	**Branches from fusion of upper, middle and lower trunks**
	Medial root of the Median Nerve (C8,T1) Medial Cutaneous nerve of the arm (C8,T1) Medial Cutaneous nerve of the Forearm (C8,T1) Ulnar nerve (C8,T1) Branch to Radial Nerve (C8,T1)	Branch to subscapularis ?Lower Subscapular (C5-8, T1) Axillary Nerve (C5-8, T1)

The axillary artery was seen to have an abnormal relationship with the median nerve. The lateral root of median crossed the artery anteriorly and met the medial root such that the median nerve lay medial to the axillary artery.

Normally the long thoracic nerve is formed from the contribution of the C5, C6 and C7 [1]. Horwartz and Tocantins have found that in 8% of the cases, C7 may fail to contribute and some times failure from contributions from C5 have been observed in dissecting laboratories [2,3]. The C5 may contribute separately to the serratus anterior muscle. In our case the long thoracic nerve is seen emerging solely from C7. There are small branches from C5 and C6 that we were unable to trace. We feel that serratus anterior may have received segmental and independent supply from these segments.

The lateral pectoral nerve in our case is seen to emerge from the posterior division of the upper trunk. Many authors have described that the lateral pectoral nerve may arise by one root from the lateral cord or by two roots from the anterior divisions of upper and middle trunks [[3,1,4], and [5]]. No case previously has described the contribution of the posterior division of the upper trunk.

The nerve to coracobrachialis is a direct branch form the lateral cord. High origin of nerve to coracobrachialis from Lateral cord is not an uncommon finding [[1,6], and [7]].

The median nerve and the musculocutaneous nerve show two communications with each other proximal to the entry of the median nerve into the coracobrachialis muscle. Communications between musculocutaneous nerves and median nerves are the most frequent of all the variations observed in the brachial plexus [8]. There are 4 kinds of communications observed between the musculocutaneous nerve and the median nerves. Out of these, Type III is the kind where communications are present distal to the entry of the musculocutaneous nerve in the coracobrachialis muscle. Our case is similar to Type III [9]. In the present study communications are also present between the lateral root of median nerve and communications of the musculocutaneous nerve.

The medial pectoral nerve in our study is a direct branch of the sixth cervical root. It is seen to give numerous branches to the pectoralis minor as it is supplying it. We were unable to study communications between the medial and lateral pectoral nerves. A case has been described wherein the medial pectoral nerve was a direct branch of the anterior division of the middle trunk [10]. We have not found findings similar to us in literature.

Upper subscapular nerve is a direct branch from the upper trunk. According to Kerr and Fazan et al the upper subscapular nerve can arise as a direct branch from the the posterior division of the upper trunk but in our case it is given off from the trunk itself [[4] and [9]].

The thoracodorsal nerve is seen as a direct branch from the sixth cervical root. Cases of it being branch of the axillary or the radial nerves are documented [3]. Our case has not been observed before.

The radial nerve is formed from the fusion of the posterior divisions of the middle and lower trunks. Only one similar case is present where the radial nerve was formed from the middle and lower trunks, the upper trunk giving no contribution to its formation [11].

The deltoid muscle is innervated directly from the brachial plexus, from the upper and middle trunks. Normally it is supplied by the axillary nerve.

The relationship of the axillary artery is not normal. In our case, the lateral root of median nerve crosses the artery anteriorly and meets the medial root such that the median nerve lies medial to the third part of axillary artery. Das and Paul have observed a similar case where there were two lateral roots of the median nerve [12]. In a study done by Pandey and Shukla on 172 cadavers, in 8 cadavers, the median nerve was formed medial to the artery and traveled as such [13]. Anomalous branches of lateral cord crossing the artery anteriorly may cause compression syndromes producing ischemia.

The subclavian and axillary system of arteries is derived from the seventh cervical intersegmental artery. Hence, the artery passes between the lateral and medial cords, representing the fifth, sixth and seventh cervical nerve on one hand and the eighth cervical and first thoracic on the other. Sometimes, the artery may arise from the sixth, the eighth or the ninth intersegmental artery and then it has abnormal relations to the plexus and the plexus is in turn modified by the presence of the abnormally placed artery. This might explain the various abnormalities seen in this case.

## Conclusion

Variations assume significance during surgical exploration of the axilla and can even fail the nerve block of infraclavicular part of the brachial plexus. Though the variations that we have mentioned here may not alter the normal functioning of the limb of the individual, it is important to keep these in mind in surgical and anaesthesiological procedures.
